# Novel approach for effective removal of methylene blue dye from water using fava bean peel waste

**DOI:** 10.1038/s41598-020-64727-5

**Published:** 2020-05-08

**Authors:** Omar S. Bayomie, Haitham Kandeel, Tamer Shoeib, Hu Yang, Noha Youssef, Mayyada M. H. El-Sayed

**Affiliations:** 1 Department of Chemistry, American University in Cairo, AUC Avenue, P.O. Box 74, New Cairo, 11835 Egypt; 2 Department of Energy and Processes, PSL Research University, Paris, France; 3 Department of Chemistry and Chemical Engineering, École Polytechnique Fédérale de Lausanne, Lausanne, Switzerland; 4 State Key Laboratory of Pollution Control and Resource Reuse, School of the Environment, Nanjing University, Nanjing, 210023 P. R. China; 5 Department of Mathematics and Actuarial Science, American University in Cairo, AUC Avenue, P.O. Box 74, New Cairo, 11835 Egypt

**Keywords:** Environmental sciences, Chemistry

## Abstract

Fava bean peels, *Vicia faba* (FBP) are investigated as biosorbents for the removal of Methylene Blue (MB) dye from aqueous solutions through a novel and efficient sorption process utilizing ultrasonic-assisted (US) shaking. Ultrasonication remarkably enhanced sorption rate relative to conventional (CV) shaking, while maintaining the same sorption capacity. Ultrasonic sorption rate amounted to four times higher than its conventional counterpart at 3.6 mg/L initial dye concentration, 5 g/L adsorbent dose, and pH 5.8. Under the same adsorbent dose and pH conditions, percent removal ranged between 70–80% at the low dye concentration range (3.6–25 mg/L) and reached about 90% at 50 mg/L of the initial dye concentration. According to the Langmuir model, maximum sorption capacity was estimated to be 140 mg/g. A multiple linear regression statistical model revealed that adsorption was significantly affected by initial concentration, adsorbent dose and time. FBP could be successfully utilized as a low-cost biosorbent for the removal of MB from wastewater via US biosorption as an alternative to CV sorption. US biosorption yields the same sorption capacities as CV biosorption, but with significant reduction in operational times.

## Introduction

Considerable amounts of synthetic dyes are used in many process industries. Textile, pharmaceutical, leather, food processing, cosmetics, and paint industries discharge into the environment about 10–15% of over 0.8 million ton of several types of dyes produced annually worldwide. It is projected that the dye business will grow annually by about 2–3% due to the increased global production and consumption of dyes. In developing countries like India and China, the production rate of dyes is increasing steadily every year. Without proper treatment, these dyes harm the environment, marine life and pose serious health threats to humans particularly upon degradation as they become toxic, recalcitrant, mutagenic, and carcinogenic^1–3^.

Methylene blue (MB) (3,7-bis(Dimethylamino)-phenothiazin-5-iumchloride) is a thiazine cationic dye commonly used for biological staining as well as coloring paper, hair, cottons and wools^4^. In addition, MB injection is used in the treatment of methemoglobinemia and urinary tract infections. However, accumulation of MB in wastewater has adverse health effects such as difficulties in breathing, vomiting, eye burns, diarrhea and nausea^5^.

Several physicochemical and biological processes have been successfully carried out in order to treat dye effluents^6^, examples of which are membrane filtration^7^, photocatalytic degradation^8^, irradiation^9^, biological treatment^10^, conventional adsorption^11^ and ultrasonic-assisted adsorption^12^. Adsorption is a commonly employed strategy due to its relative facile operation, availability of its adsorbents, selectivity and potential for scale-up. Among employed adsorbents, activated carbon is commonly used due to its efficiency and high surface area^13^. However, it has substantial limitations due to its high cost of regeneration and its impact on the environment. Performance of the adsorption process could be improved via ultrasonication. The ultrasonication process produces waves that enhance and accelerate mass transfer by generating shear forces in the solution via micro-streaming, micro-turbulence, acoustic waves, and micro jets without significantly changing the equilibrium of the adsorption/desorption system. This, in turn, could speed up the adsorption^14,15^.

For the sake of environmental sustainability and for economic reasons, many researchers investigated biosorption as a low-cost, safe and potentially effective alternative to traditional sorption; specifically for the removal of heavy metals and dyes from wastewater^16–18^. Several studies reported MB removal using untreated biosorbents or those treated thermally and/or activated using acids or bases. For studies that utilized untreated biosorbents, some of the highest maximum adsorption capacities reported (18–333 mg/g) pertained to cellulose- and lignin-based adsorbents such as chitin^19^, rice straw lignin^20^, wood cherry tree^14^, wheat shells^21^, as well as fruit peels of banana, orange and pineapple^22^ in addition to biomass such as *Aspergillus fumigatus*^23^ and *Bacillus subtilis*^24^, and others like fly ash geopolymer^25^, white pine sawdust^18^, and *Abelmoschus esculentus* seeds^26^. As for pre-treated adsorbents, some of the highest maximum adsorption capacities (20–672 mg/g) were reported for walnut wood activated carbon treated with nitric acid^27^, spent tea modified by NaOH^28^, sulfuric-acid activated cotton stalk^29^, base-activated bamboo charcoal^30^, HCl-activated oil palm fiber^31^, and coconut husk activated carbon modified by KOH at 816 °C^32^, in addition to pre-treated peels such as oven-dried *Artocarpus camansi* peels^33^, banana peels activated with NaOH^34^, jackfruit peel modified with microwave induced NaOH activation^35^, and pomelo skin activated by NaOH using microwave heating^36^.

Fava beans (*Vicia faba*) are highly consumed in different parts of the world like the Mediterranean area in Europe and Africa, Latin America, China and India, thus massive amounts of the peels are disposed of as waste. Only a portion of this waste is used by farmers as animal feedstock. In a previous study, fava bean powder was used for the removal of heavy metal ions of Pb(II), Cd(II) and Zn(II)^37^ to achieve removal efficiencies of 100%, 92.86% and 36.86%, respectively. Broad bean peels, on the other hand, were utilized to remove MB dye with a maximum adsorption capacity of 192.7 mg g^−1^ at 30 °C^38^.

The objective of the present study is to develop a viable and efficient biosorption process for the removal of MB dye from aqueous solutions using fava bean peels (FBP) as low-cost adsorbents. For that purpose, biosorption was conducted using ultrasonic-assisted (US) shaking as an alternative to conventional (CV) magnetic stirring. Sorption performance, in terms of capacity and rate, was evaluated and compared to that of CV biosorption under various conditions including adsorbent dosage, initial dye concentration, pH and contact time. A statistical multiple linear regression model was developed using the backward method on R program to determine the influencing operating parameters.

## Materials and methods

### Materials

MB was purchased from Central Drug House (CDH, India), with λ_max_ of 663–667 nm. The dye has a pK_a_ of 3.8, hence is neutral at pH ≤ 3.8 and positively-charged above pH 3.8. FBP were obtained from a local market in Cairo, and were ground in a Braun blender and sieved to a particle size range of 0.25 to 2 mm, then washed with tap water to remove solid impurities and dust. The peels were then washed with distilled water to remove any adsorbed particles, left to dry out in a petri dish, then sealed and stored for future use. The composition of Egyptian FBP is 46.5% carbohydrates, 25.2% proteins, 10.3% dietary fibers and 1.5% lipids^38^.

### Characterization

The chemical structure of FBP was analyzed using the KBr pellet method onto a Thermo Scientific Nicolet 380 Fourier Transform Infrared (FTIR) spectrometer, which houses an EverGlo lamp that emits infrared radiation in the spectral range from 7800 to 350 cm^−1^. Surface morphology of FBP before and after sorption was also studied using LEO Field Emission Scanning Electron Microscope (SEM) employed at 1 nm resolution and a magnification of 1000×. Dynamic light scattering was conducted using Malvern Nano-ZS90 to measure the particle size distribution before and after ultrasonication for the maximum operation time of 70 min. Brunauer-Emmett-Teller (BET) measurements were performed on a BETASP 2020 analyzer, and the nitrogen adsorption isotherm values were determined using an ASAP 2020-Micromeretics apparatus at 77 K. The time required to attain sorption or desorption equilibrium was between 15 and 20 minutes.

### Kinetic studies

Batch kinetic experiments were performed using CV and US shaking. In each mode of operation, initial concentrations of MB (3.6, 7, 13, 25, 50, 75 and 100 mg/L) were prepared by simply weighing the desired amount and dissolving it in 100 mL of distilled water. The runs were conducted in 250-mL glass conical flasks in which the desired mass of adsorbent (0.1, 0.25, 0.5, 0.75 and 1g) was added to 100 mL of the MB solution. The experiments took place at room temperature (27 ± 2 °C), and at different initial pH values of 5.8, 7.2 and 9.2, adjusted using 0.1 N NaOH. CV experiments were conducted by shaking the conical flasks on a Burrell Wrist Action Shaker Model 75 shaker set to 416 rpm, whereas the US experiments were undertaken using an ultrasonicator (model VWR® Symphony™ Ultrasonic Cleaners 150HT) which operates at a frequency of 35 KHz. Each conical flask was left to shake for a specific time duration after which it was centrifuged at 7500 rpm for 10 minutes. CV biosorption durations were 2, 5, 10, 15, 20, 25, 30 and 40 minutes, while those of US biosorption were 2.5, 5, 7.5, 10, 12.5, 15, 17.5 and 20 minutes due to the faster kinetics achieved under sonication. Adsorption capacities were calculated according to the following equation:

1 $$q=({C}_{o}\,-\,C)V/W$$

where *q* is the amount adsorbed at time *t*, *C* is the concentration of the dye in solution at time *t*, *C*_o_ is the initial concentration of the dye, *V* is the volume of the solution and *W* is the dry weight of the biosorbent.

The percentage removal was calculated using:

2 $$ \% R=(({C}_{o}\,-\,C)/{C}_{o})\ast 100$$

Kinetics of adsorption was examined using the pseudo-second order kinetic model, expressed by the following linearized form^17,39^

3 $$\frac{t}{q}=\frac{1}{k{q}_{e}^{2}}+\frac{t}{{q}_{e}}$$

where *k* is the rate constant and *q*_*e*_ is the amount adsorbed at equilibrium.

To study sorption equilibrium and to determine the maximum sorption capacity (*q*_*m*_), sorption experiments were conducted in a manner similar to that described for kinetic studies. However, sorption was carried out for 24 h at 27 ± 2 °C and pH of 5.8 using different initial concentrations. Sorption isotherms were constructed as *q*_*e*_ versus *C*_*e*_ and were then fitted to Langmuir and Freundlich models whose equations can be expressed by the following linear forms (Eqs. 4 and 5, respectively)^40^

4 $$\log \,{q}_{e}=\,\log \,{K}_{f}+\frac{1}{n}log{C}_{e}$$

5 $$\frac{{C}_{e}}{{q}_{e}}=\frac{1}{{q}_{m}b}+\frac{{C}_{e}}{{q}_{m}}$$

*K*_*f*_ and *n* are parameters specific for each adsorbent, while *q*_*m*_ is the maximum sorption capacity which can be calculated from the slope of Eq. 5, and *b* is Langmuir constant.

### Statistical analysis

Multiple linear regression was used to analyze the experimental results employing the program R 3.5.1. The most significant factors affecting the amount adsorbed at equilibrium (*q*_*e*_) were selected via the backward method.

In general, the dependent variable (*q*_*e*_) is expressed as follows

6 $${q}_{e}={\beta }_{0}+{\beta }_{1}{X}_{1}+{\beta }_{2}{X}_{2}+\ldots +{\beta }_{n}{X}_{n}+{\epsilon }$$

where ${\beta }_{1},{\beta }_{2},\ldots ,{\beta }_{n}$ are the regression coefficients, ${X}_{1},{X}_{2},\ldots ,{X}_{n}$ are the factors affecting *q*_*e*_, ${\epsilon }$ are the random errors due to other factors not included in the study.

## Results and discussion

### Biosorption indicators

**Figure 1:**
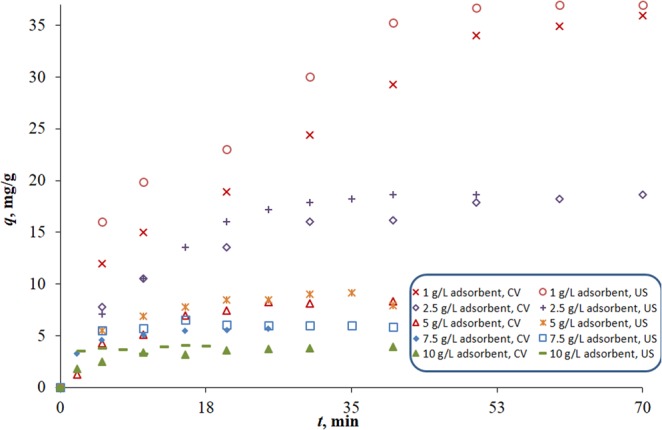
Uptake profiles for the CV versus the US biosorption of MB onto FBP. Sorption conditions are 50 mg/L initial concentration and pH 5.8 at different adsorbent doses.

In this section, the effect of different operating parameters (time, initial concentration, adsorbent dose and pH) on two sorption indicators (equilibrium sorption capacity *q*_*e*_ and % removal) is investigated. To do so, the sorption time profiles for the uptake of MB (*q*) onto FBP using CV and US shaking were first constructed under different operating conditions (Figs. 1 and S1–S3, supplementary material). To investigate the effect of adsorbent dose, the uptake profiles of 50 mg/L MB onto different doses of FBP at pH 5.8 were plotted in Fig. 1. In each of these profiles (Fig. 1), the effect of time on the adsorption capacity is manifested. Initially, the capacity increases rapidly with time then it flattens out as equilibrium is approached. Equilibrium time varies between 20 and 40 min depending on the adsorbent dose and mode of operation. Similar profiles were constructed to examine the effect of initial concentration at pH 5.8 using 5 g/L FBP at low (3.6–13 mg/L) and high (25–100 mg/L) MB concentration ranges (Figs. S1 and S2, respectively). Similarly, the effect of pH was studied for 50 mg/L MB onto 5 g/L FBP at different pH values (Fig. S3). For all profiles, *q* increased with time until it flattened out when *q*_*e*_ was approached.

By comparing the CV and the US uptake profiles at the same operating conditions, it can be observed that they have similar shapes as well as comparable *q*_*e*_ values (*p* > 0.05). The US profiles however, have higher initial rates as observed in their steeper initial slopes allowing them to reach equilibrium at a shorter time.

**Figure 2:**
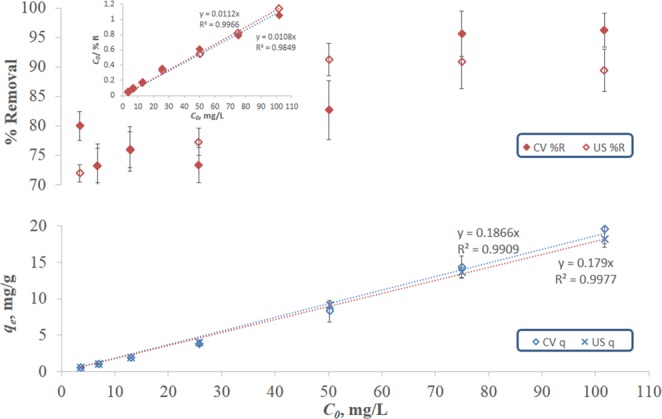
Maximum percentage removal efficiency and equilibrium uptake capacity of MB onto FBP as a function of initial concentration. Removal was achieved using CV and US biosorption at pH 5.8 with an adsorbent dose of 5 g/L. Inset shows the linear forms of the % *R*-*C*_0_ hyperbolic relations.

To study the effect of operating parameters on the sorption, values of *q*_*e*_ and % removal were obtained from each sorption profile and were plotted versus each varying parameter. The effect of initial dye concentration is depicted in Fig. 2, which shows that *q*_*e*_ increases linearly with increasing the initial concentration under both CV and US conditions. This behavior has been previously reported for the sorption of various heavy metals and dyes onto biosorbents, and it can be ascribed to an increase in the driving force which overcomes the mass transfer resistance^17,41^. As for the % removal, it increases with concentration in a hyperbolic manner until it flattens out and remains almost constant at high concentrations. Similar behavior was reported for the biosorption of lead (5–100 mg/L) onto peach/apricot stones^42^; and was attributed to the saturation of sorption active sites. Comparing the *q*_*e*_ versus *C*_0_ profiles for CV as opposed to US biosorption, it can be inferred that ultrasonication did not significantly affect the value of *q*_*e*_. A similar conclusion can be drawn for the % *R* versus *C*_0_ profiles. The linearized forms of the hyperbolic relations for the CV and US profiles are shown in the inset of Fig. 2, and the corresponding hyperbolic equation for both profiles can be expressed as follows:

7 $$ \% R=\frac{a\times {C}_{0}}{(b+{C}_{0})}$$

where *a* is the maximum % removal and *b* is the *C*_0_ corresponding to 50% removal. Values of *a* and *b* for CV biosorption are not significantly different from those of US biosorption.

**Figure 3:**
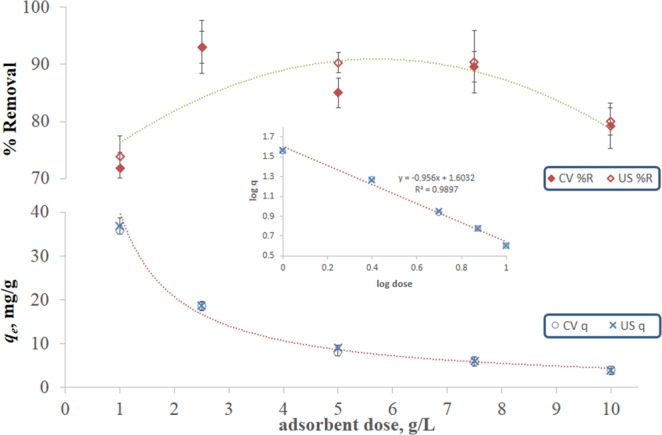
Maximum percentage removal efficiency and equilibrium uptake capacity of MB onto FBP as a function of adsorbent dose. Removal was achieved using CV and US biosorption at pH 5.8 at an initial concentration of 50 mg/L. Inset shows the linear forms of the *q*_e_- adsorbent dose relations.

Figure 3 illustrates the effect of adsorbent dose on biosorption indicators, where *q*_*e*_ for both CV and US biosorption is shown to decrease logarithmically with increasing adsorbent dose. The logarithmic relation is evident from the figure inset. Similar behavior was observed for biosorption of Cr (III) onto orange peel wastes and for that of Pb (II), Cd (II) and Cu (II) onto olive pomace wastes. This could be owed to the reduction in surface area caused by the aggregation of particles and overlapping of sorption active sites at the high adsorbent dose^17^. Percent removal, on the other hand, increases with increasing adsorbent dose until it reaches a maximum at about 5 g/L dose, after which it declines. This behavior was previously reported for biosorption of Cu (II) onto antibiotic waste as well as sorption of Pb (II) onto solid waste produced from olive-oil industry^17,43^ and the decline in % removal was due to the saturation of active sites. Corresponding plots for pH are not shown since no statistical difference (*p* > 0.05) according to t-test was obtained in the values of the equilibrium uptake capacity or % removal with change in the pH of the solution.

### Kinetic parameters for biosorption

To quantitatively assess the effect of operating conditions on sorption rates, the kinetic profiles alluded to earlier were fitted to the pseudo-second order rate model and values of the relevant kinetic rate constants were determined and presented in Table 1. The predicted profiles were found to be in good agreement with their experimental counterparts as evident from the high correlation coefficient (*R*^2^) values compiled in Table 1.

**Table 1:** Pseudo second-order (*k*) kinetic rate constants for conventional and ultrasonic biosorption of MB onto FBP. Sorption conditions for section A: 5 g/L adsorbent dose, pH 5.8 and different initial concentrations, section B: 50 mg/L initial concentration, pH 5.8 and different adsorbent doses. Correlation factors are also included in the table.

	*C*_0_ *mg/L*	*k (CV)* (g/mg.min)	*R*^2^	*k (US)* (g/mg.min)	*R*^2^	*% increase in k*
**Section A**	3.6	0.97 ± 0.09	0.997	4.00 ± 0.32	0.992	312
	7.0	0.48 ± 0.04	0.979	1.86 ± 0.19	0.997	288
	13	0.15 ± 0.01	0.996	0.52 ± 0.04	0.998	254
	25	0.10 ± 0.01	0.985	0.35 ± 0.03	0.998	246
	50	0.018 ± 0.001	0.996	0.051 ± 0.005	0.995	183
	75	0.017 ± 0.001	0.999	0.030 ± 0.002	0.999	79
	101	0.012 ± 0.006	0.999	0.016 ± 0.001	0.998	34
**Section B**	***Adsorbent Dose (g/L)***	***k (CV) (g/mg.min)***	***R***^**2**^	***k (US) (g/mg.min)***	***R***^**2**^	***% increase in k***
	1.0	0.0015 ± 0.0001	0.997	0.0015 ± 0.0002	0.996	0
	2.5	0.0040 ± 0.0004	0.997	0.0040 ± 0.0003	0.995	0
	5.0	0.018 ± 0.001	0.987	0.051 ± 0.004	0.975	183
	7.5	0.097 ± 0.007	0.998	0.315 ± 0.020	0.999	225
	10	0.102 ± 0.005	0.999	0.398 ± 0.039	0.984	290
**Section C**	***pH***	***k (CV)*** **(g/mg.min)**	***R***^***2***^	***k (US)*** **(g/mg.min)**	***R***^***2***^	***% increase in k***
	2.8	0.013 ± 0.001	0.996	0.017 ± 0.002	0.995	31
	3.8	0.026 ± 0.004	0.999	0.037 ± 0.004	0.994	42
	5.1	0.034 ± 0006	0.999	0.061 ± 0.006	0.992	79
	5.8	0.018 ± 0.005	0.996	0.051 ± 0.005	0.995	183
	7.2	0.066 ± 0.006	0.999	0.091 ± 0.006	0.994	38
	7.9	0.065 ± 0.006	0.998	0.087 ± 0.006	0.994	34
	9.2	0.068 ± 0.008	0.999	0.086 ± 0.008	0.998	26

**Figure 4:**
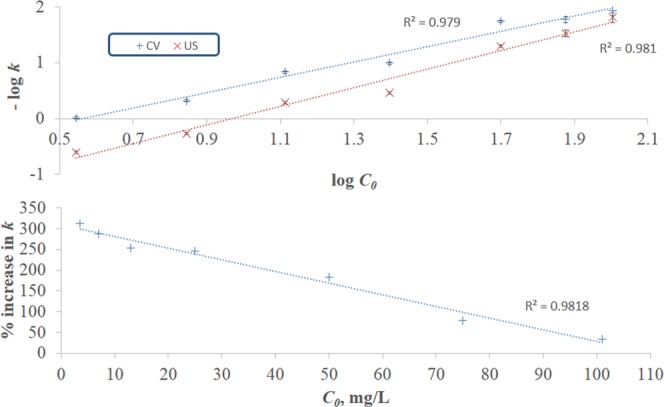
CV and US kinetic rate constants (top panel), and % increase in the rate constants with ultrasonication (bottom panel) as a function of initial concentration.

Section A of Table 1 presents the variation in *k* with initial concentration. This section of the table along with Fig. 4 (top panel) show that *k* varies inverse proportionally with *C*_0_ in a logarithmic manner. The same trend is observed for both CV and US biosorption. Reduction in *k* and hence in rate could be a result of the increased diffusional resistance across the boundary layer. Percentage increase in *k* as a result of ultrasonication is also shown in section A of Table 1 and Fig. 4 (bottom panel) which display that the percentage increase drops with increasing *C*_0_ in a linear manner. This indicates that the effect of ultrasonication in enhancing the rate is mitigated at the higher concentrations, where the mass transfer resistance is reduced by virtue of the high concentration gradient driving force regardless of ultrasonication and hence the influence of ultrasonication is not highly pronounced. The highest % increase in *k* recorded at the low concentration range (3.6–25 mg/L) corresponds to % removal of 70–80%. The advantage that ultrasonication provides at this low-concentration range is particularly important from the practical point of view since removing dyes in this concentration range is challenging, although various dye effluents exist in this range^44^.

The effect of adsorbent dose on the kinetic rate constants is shown in section B of Table 1, where it can be deduced that *k* increases logarithmically with increasing the dose (i.e. the plot of log *k*-log dose is linear). This could be due to the increase in the number of vacant sites encountered as the amount of adsorbent increases. The rate, being proportional to the number of vacant sites, consequently increases. The percentage increase in the value of *k* relative to that obtained through CV was determined at each adsorbent dose in order to investigate the effect of ultrasonication (Table 1, section B). At low adsorbent doses, values of *k* for CV and US biosorption are not statistically different. However, with higher doses, *k* is significantly enhanced with ultrasonication and this enhancement becomes more pronounced as the dose increases. One plausible explanation could be that localized high temperatures caused by ultrasonic waves produce cavities with more exposed functional groups (vacant active sites) in the large particles.

**Figure 5:**
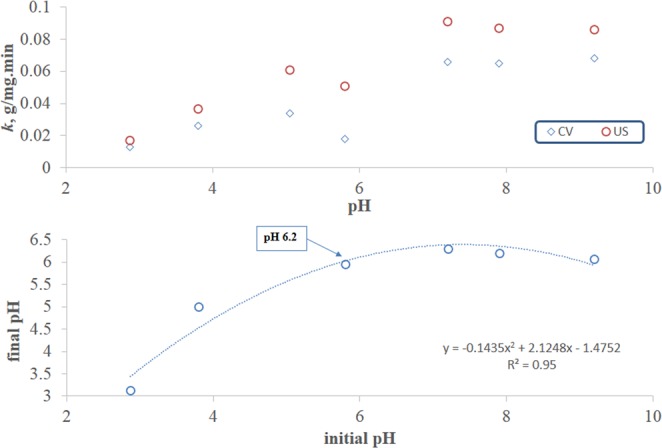
Rate constants for sorption of 50 mg/L MB onto 5 g/L FBP at different pH values (top panel) and determination of PZC (bottom panel).

The data presented in Table 1, section C along with Fig. 5 (top panel) also show that for both CV and US biosorption, *k* increased with the first three pH values (2.8, 3.8 and 5.1) at which measurements were obtained. In both cases, *k* significantly decreased at about pH 6 then increased significantly to reach their respective maximum values at about pH 7.2 where further increases in pH up to a value of 9.2 did not show significant changes in *k*.

The point of zero net charge (PZC) which corresponded to pH 6.2 was determined by plotting the final versus the initial pH values and determining the point at which they equate as shown in Fig. 5 (bottom panel). It is interesting to note that the pH value corresponding to the PZC is close to that at which the lowest observable *k* value was obtained in both biosorption modes. This is probably due to the minimal electrostatic attraction between the zero-charged fava peels and the positively-charged MB (PZC > pK_a_ of dye). At pH values below PZC and above pK_a_ of the dye, relatively higher values of *k* were obtained in both cases despite the electrostatic repulsion between the positively charged adsorbent and positively charged dye. This implies that the adsorbent-dye interaction is not purely electrostatic. Above PZC, *k* increases with pH due to the electrostatic attraction between the positively-charged dye and the negatively-charged adsorbent. This behavior again indicates that the interaction involves both electrostatic and non-electrostatic binding. The latter could be van der Waal’s, dipole interactions or chemical bonding. Furthermore, the highest %increase in *k* due to ultrasonication is observed near PZC. Despite the net zero charge on the adsorbent at PZC, ultrasonic waves could have induced partial charge on the surface of the produced cavities and thus enhanced sorption.

### Biosorption under equilibrium conditions

The equilibrium isotherm for the CV sorption of MB onto FBP was fitted to each of the Langmuir and Freundlich models (Fig. S4), where it was shown to be better described using the Langmuir model as indicated by the higher value of the correlation factor (*R*^2^ = 0.9636) relative to that of Freundlich (*R*^2^ = 0.9097). It is to be noted here that the *q*_*m*_ obtained under equilibrium conditions (140 mg/g) was not attained in the kinetic studies presented earlier. This could be due to the much longer operational time of the equilibrium study (24 h) that has allowed for further adsorption to take place through pore diffusion mechanism. Values of *q*_*m*_ reported in previous literature for other treated and untreated fruit peels are presented in Table 2. By comparing these values to that obtained in this study, it can be inferred that FBP are superior to most of the other untreated peels. In all of these studies, the capacities of untreated peels ranged from 18 to 133 mg/g except for one study which reported a capacity of 333 mg/g for melon peels. However, FBP have lower performance relative to some of the treated ones whose capacities ranged from 19.7 to 409 mg/g and the activated carbons derived from fruit peels which ranged from 400–1193 mg/g. Nevertheless, working with untreated peels could provide a more cost effective and greener option than working with treated peels. This is yet to be investigated through looking into various technical and economic parameters like, for instance, performance of the treated versus the untreated peels, cost of materials, equipment and energy involved in the treatment process, recovery of the treatment solvents, in addition to the environmental impact of the treatment process.

**Table 2:** Summary of the recent studies performed on MB removal using fruit peels and their activated forms.

Adsorbent peels	*q*_m_, mg/g	pH/T/PS^*^	Reference
Untreated FBP	140	5.8/27/0.25-2	This work
Untreated orange peel	24.0	7.2/30/ <5	^45^
Untreated banana peel	18.0	7.2/30/ <5	^45^
Untreated dragon fruit peels	62.6	7/30/−	^46^
Untreated pineapple peels	97.1	6/30/0.355-0.5	^22^
Untreated cucumber peels	111.1	7/20/0.500	^47^
Untreated melon peels	333.3	−/25/0.5-1	^48^
Untreated oak acorn peel	120.4	7/24/0.5	^49^
Untreated potato peels	33.8	8/20/0.020	^50^
Untreated potato peels	105.2	7/25/0.5–1.25	^51^
Untreated Pomelo peels powder	133	30/8/−	^52^
Oven dried *Artocarpus camansi* peels	409	6/25/0.355–0.850	^33^
NaOH-activated banana peels	19.7	5/20/0.315	^34^
NaOH – treated rambutan peel	231.3	−/30/−	^53^
Activated jackfruit peel (AC^**^) (NaOH / microwave heating)	400	−/30/1–2	^35^
Pomelo skin (AC) (NaOH/microwave heating)	501.1	12/30/1–2	^36^
Mangosteen peel waste (AC) (ZnCl_2_/high temperature activation)	1193	9/25/−	^13^

^*^T: temperature (°C), PS: particle size in mm.

^**^AC: activated carbon.

### Characterization of FBP pre- and post biosorption

The FTIR spectra of the FBP before and after CV and US sorption (Fig. S5) showed a peak at 3500 cm^−1^ that could be ascribed to the OH stretching vibration, in addition to a peak at 1700 cm^−1^ that could be attributed to the amide carbonyl stretch^18^,^33^,^34^. Comparing the IR spectra pre-adsorption to those post-adsorption under CV or US agitation, it can be concluded that both peaks were shifted to the left after adsorption. This indicates the formation of higher energy bonds which consequently implies binding as a result of adsorption. Possible electrostatic interaction between the positively charged nitrogen or sulfur on MB and the negatively charged lone pair on the carbonyl oxygen of FBP could have occurred, or alternatively a redox reaction. Additionally, hydrogen bonds may have been formed between the amine groups of the dye and hydroxyl groups of FBP. Furthermore, the position of the peaks pertaining to CV and US biosorption remained unchanged, indicating that ultrasonication did not decompose or chemically alter the structure of the peels and therefore ultrasonication could be used as an alternative to conventional agitation.

Surface morphology of FBP before and after biosorption is also depicted in Fig. S6 where it can be seen that the surface of the peels became rougher after adsorption of MB. In addition, no change in the morphology can be observed with ultrasonication of FBP. Furthermore, the hydrodynamic diameter of FBP before and after ultrasonication was measured to be 2520 ± 764 and 2406 ± 753 nm, respectively implying that ultrasonication had no significant effect on particle size. This data along with the FTIR measurements suggest that the structure of FBP was not damaged by the process performed.

**Figure 6:**
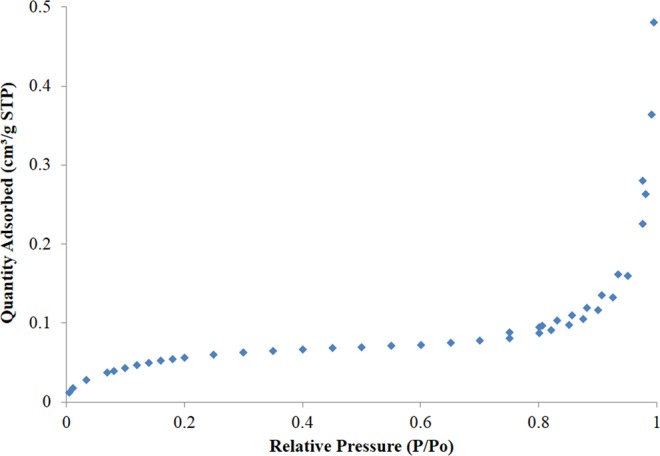
BET type II isotherm for FBP describing strong interaction to macroporous adsorbents.

**Figure 7:**
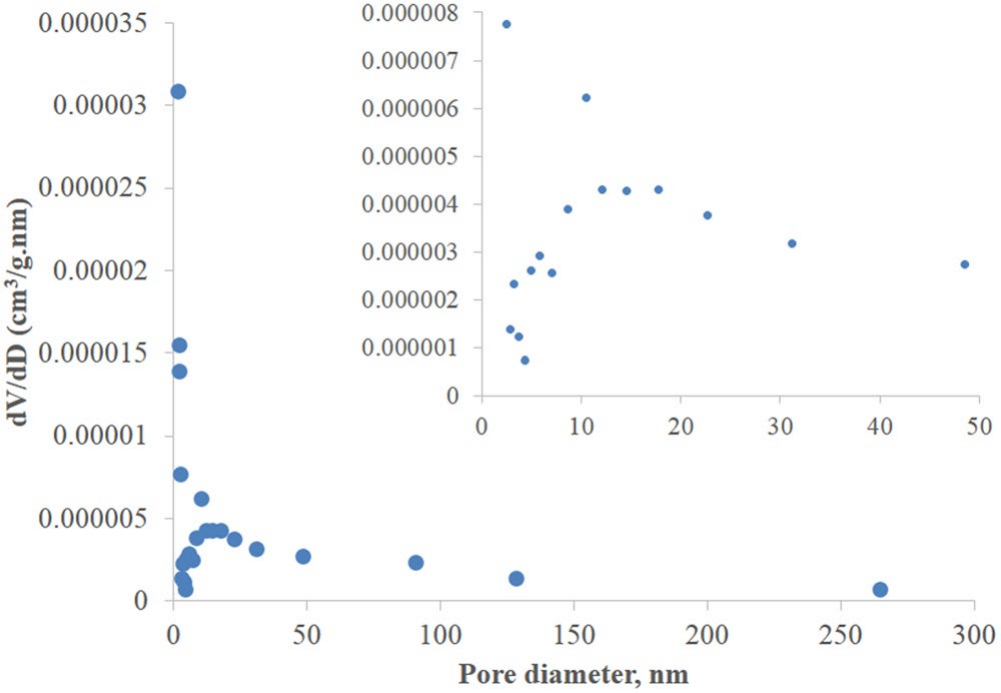
BET isotherm for pore size distribution with magnified inset for clarity.

The BET adsorption isotherm for FBP is depicted in Fig. 6. According to IUPAC, the isotherm is Type II which describes strong interaction pertaining to macroporous adsorbents. A wide pore size distribution ranging from 1.7 to 264 nm pore diameter was obtained as shown in Fig. 7, which indicates that some mesopores also exist along with macropores. In addition, the total surface area was found to be 0.2108 ± 0.0035 m²/g, while the total pore volume was 0.00067 cm^3^/g.

### Predicting the sorption capacity via statistical modeling

In order to run the regression analysis, a correlation study was performed to examine if the linear regression analysis can be applied. The variables or factors included in the study were the initial concentration, *C*_0_, adsorbent dose, time, *t*, pH and the stirring method, *X*. *X* was defined as a binary variable which takes the value 0, if CV or the value 1 if US. The correlation analysis revealed that *q*_*e*_ is significantly correlated with *C*_0_, *t*, and dose and hence regression analysis was carried out. All variables were included at the first stage and then the backward method was performed to select the best set of factors affecting *q*_*e*_. The pH factor was dropped because it was not significant in presence of the other factors in the equation, thus the final model has the following form

8 $$predicted\,{q}_{e}=20.41+0.16{C}_{0}+0.15t-13\,ln(dose)+2.5\,X$$

The model developed after dropping the pH is highly significant with a *p*-value of 1.074e-15. The adjusted *R*^*2*^ is 91% which means that the chosen factors explain 91% of the variation in *q*_*e*_. This strong relation could be clearly shown (Fig. S7) by comparing the predicted *q*_*e*_ values to their corresponding experimental values.

## Conclusion

CV and US biosorption were successfully utilized for the removal of MB by FBP under different conditions. A multiple linear regression statistical model revealed that initial concentration, adsorbent dose and time were influencing factors. The US process is recommended since it provides faster removal than CV while achieving the same maximum sorption capacity and maintaining the chemical structure of the adsorbent. Future work will consider optimizing the regeneration process for the exhausted FBP, and validating the cost effectiveness and potential of scale-up of the US process. This proof-of-concept study could also be extended to other contaminants of emerging concern.

## Supplementary information

Supplementary material.

## References

[1] Karaer H, Kaya I (2016). Synthesis, characterization of magnetic chitosan/active charcoal composite and using at the adsorption of methylene blue and reactive blue 4. Microporous Mesoporous Mater.

[2] Hassan MA, El-Nemr A (2017). Health and environmental impacts of dyes, minireview. AJESE.

[3] Dyes, https://ihsmarkit.com/products/dyes-chemical-economics-handbook.html (2018).

[4] Gupta VK, Imran A, Saini VK (2004). Removal of Rhodamine B, Fast Green, and Methylene Blue from wastewater using red mud, an Aluminum Industry Waste. Ind Eng Chem Res.

[5] Mahapatra K, Ramteke DS, Paliwal LJ (2012). Production of activated carbon from sludge of food processing industry under controlled pyrolysis and its application for methylene blue removal. J Anal Appl Pyrolysis.

[6] Hor KY (2016). Evaluation of physicochemical methods in enhancing the adsorption performance of natural zeolite as low-cost adsorbent of methylene blue dye from wastewater. J Clean Prod.

[7] Zinadini S (2014). Novel high flux antifouling nanofiltration membranes for dye removal containing carboxymethyl chitosan coated Fe3O4 nanoparticles. Desalination.

[8] Kerkez-Kuyumcu Ö (2015). A comparative study for removal of different dyes over M/TiO2 (M = Cu, Ni, Co, Fe, Mn and Cr) photocatalysts under visible light irradiation. J Photochem Photobiol A Chem.

[9] Son G, Lee H (2016). Methylene blue removal by submerged plasma irradiation system in the presence of persulfate. Environ Sci Pollut Res.

[10] Kumar AN, Reddy CN, Mohan SV (2015). Biomineralization of azo dye bearing wastewater in periodic discontinuous batch reactor: Effect of microaerophilic conditions on treatment efficiency. Bioresour Technol.

[11] Elwakeel KZ, Elgarahy AM, Mohammad SH (2017). Use of beach bivalve shells located at Port Said coast (Egypt) as a green approach for methylene blue removal. J Environ Chem Eng.

[12] Asfaram A, Ghaedi M, Hajati S, Goudarzi A (2016). Synthesis of magnetic γ-Fe2O3 -based nanomaterial for ultrasonic assisted dyes adsorption: Modeling and optimization. Ultrason Sonochem.

[13] Nasrullah A (2019). Mangosteen peel waste as a sustainable precursor for high surface area mesoporous activated carbon: Characterization and application for methylene blue removal. J Clean Prod.

[14] Ardekani PS, Karimi H, Ghaedi M, Asfaram A, Purkait MK (2017). Ultrasonic assisted removal of methylene blue on ultrasonically synthesized zinc hydroxide nanoparticles on activated carbon prepared from wood of cherry tree: Experimental design methodology and artificial neural network. J Mol Liq.

[15] Khafri HZ, Ghaedi M, Asfaram A, Safarpoor M (2017). Synthesis and characterization of ZnS:Ni-NPs loaded on AC derived from apple tree wood and their applicability for the ultrasound assisted comparative adsorption of cationic dyes based on the experimental design. Ultrason Sonochem.

[16] Daneshvar E, Vazirzadeh A, Sillanpää M, Bhatnagar A (2017). A comparative study of methylene blue biosorption using different modified brown, red and green macroalgae – Effect of pretreatment. Chem Eng J.

[17] El-Sayed, H. E. M. & El-Sayed, M. M. H. Assessment of Food Processing and Pharmaceutical Industrial Wastes as Potential Biosorbents: A Review. *BioMed Research International* (2014). 10.1155/2014/146769 PMC4109414 25110656

[18] Salazar-Rabago JJ, Leyba-Ramos R, Rivera-Utrilla J, Ocampo-Perez R, Cerino-Cordova FJ (2017). Biosorption mechanism of Methylene Blue from aqueous solution onto White Pine (Pinus durangensis) sawdust: Effect of operating conditions. Sustain Environ Res.

[19] Dotto GL (2015). Adsorption of Methylene Blue by ultrasonic surface modified chitin. J Colloid Interface Sci.

[20] Zhang S, Wang Z, Zhang Y, Pan H, Tao L (2016). Adsorption of Methylene Blue on Organosolv Lignin from Rice Straw. Procedia Environ Sci.

[21] Bulut Y, Aydin H (2006). A kinetics and thermodynamics study of methylene blue adsorption on wheat shells. Desalination.

[22] Krishni RR, Foo KY, Hameed BH (2014). Food cannery effluent, pineapple peel as an effective low-cost biosorbent for removing cationic dye from aqueous solutions. Desalin Water Treat.

[23] Abdallah R, Taha S (2012). Biosorption of methylene blue from aqueous solution by nonviable Aspergillus fumigatus. Chem Eng J.

[24] Tural, B., Ertaş, E., Enez, B., Fincan, S. A. & Tural, S. Preparation and characterization of a novel magnetic biosorbent functionalized with biomass of Bacillus Subtilis: Kinetic and isotherm studies of biosorption processes in the removal of Methylene Blue. *J Environ Chem Eng* **5** (2017).

[25] Novais RM, Ascensão G, Tbaldi DM, Seabra MP, Labrincha JA (2018). Biomass fly ash geopolymer monoliths for effective methylene blue removal from wastewaters. J Clean Prod.

[26] Nayak AK, Pal A (2017). Green and efficient biosorptive removal of methylene blue by Abelmoschus esculentus seed: Process optimization and multi-variate modeling. J Environ Manage.

[27] Ghaedi M, Mazaheri H, Khodadoust S, Hajati S, Purkait MK (2015). Application of central composite design for simultaneous removal of methylene blue and Pb2+ ions by walnut wood activated carbon. Spectrochim Acta Part A Mol Biomol Spectrosc.

[28] Nasuha N, Hameed BH (2011). Adsorption of methylene blue from aqueous solution onto NaOH-modified rejected tea. Chem Eng J.

[29] Deng H, Lu J, Li G, Zhang G, Wang X (2011). Adsorption of methylene blue on adsorbent materials produced from cotton stalk. Chem Eng J.

[30] Hameed BH, Din ATM, Ahmad AL (2007). Adsorption of methylene blue onto bamboo-based activated carbon: Kinetics and equilibrium studies. J Hazard Mater.

[31] Ofomaja AE (2007). Kinetics and mechanism of methylene blue sorption onto palm kernel fibre. Process Biochem.

[32] Tan IAW, Ahmad AL, Hameed BH (2008). Adsorption of basic dye on high-surface-area activated carbon prepared from coconut husk: Equilibrium, kinetic and thermodynamic studies. J Hazard Mater.

[33] Lim LBL (2017). Breadnut peel as a highly effective low-cost biosorbent for methylene blue: Equilibrium, thermodynamic and kinetic studies. Arab J Chem.

[34] Amel, K., Hassen, M. A. & Kerroum, D. Isotherm and kinetics study of biosorption of cationic dye onto banana peel. *Energy Procedia*, 286-295 (2012).

[35] Foo KY, Hameed BH (2012). Potential of jackfruit peel as precursor for activated carbon prepared by microwave induced NaOH activation. Bioresour Technol.

[36] Foo KY, Hameed BH (2011). Microwave assisted preparation of activated carbon from pomelo skin for the removal of anionic and cationic dyes. Chem Eng J.

[37] Etorki AM, El-Rais M, Mahabbis MT, Moussa NM (2014). Removal of some heavy metals from wastewater by using of fava beans. Am J Anal Chem.

[38] Hameed BH, El-Khaiary MI (2008). Sorption kinetics and isotherm studies of a cationic dye using agricultural waste: Broad bean peels. J Hazard Mater.

[39] Mostafa, A.A., El-Sayed, M.M.H., Mahmoud, A.A., Gamal-Eldeen, A.M., Bioactive/natural polymeric scaffolds loaded with ciprofloxacin for treatment of osteomyelitis, *AAPS Pharm Sci Tech* 1-14 (2016). 10.1208/s12249-016-0605-0 27520562

[40] Sorour MH, Hani HA, Shaalan HF, El- Sayed MMH (2016). Experimental screening of some chelating agents for calcium and magnesium removal from saline solutions. Desalin Water Treat.

[41] El-Sayed MMH, El-Sayed HEM, Ali SS (2014). Cadmium removal from aqueous solutions via biosorption onto acclimatized activated sludge. IDA J Desalin Water Reuse.

[42] Rashed MN (2006). Fruit stones from industrial waste for the removal of lead ions from polluted water. Environ Monit Assess.

[43] Blazquez G, Calero M, Hernainz F, Tenorio G, Martin-Lara MA (2010). Equilibrium biosorption of lead(II) from aqueous solutions by solid waste from olive-oil production. Chem Eng J.

[44] Yaseen DA, Scholz M (2019). Textile dye wastewater characteristics and constituents of synthetic effluents: a critical review. Int J Environ Sci Technol.

[45] Annadurai G, Juang R, Lee D (2002). Use of cellulose-based wastes for adsorption of dyes from aqueous solutions. J Hazard Mater.

[46] Mallampati R, Xuanjun L, Adin A, Valiyaveettil S (2015). Fruit peels as efficient renewable adsorbents for removal of dissolved heavy metals and dyes from water. ACS Sustain. Chem. Eng..

[47] Akkaya G, Güzel F (2014). Application of some domestic wastes as new low-cost biosorbents for removal of methylene blue: kinetic and equilibrium studies. Chem. Eng. Commun..

[48] Djelloul C, Hamdaoui O (2015). Dynamic adsorption of methylene blue by melon peel in fixed- bed columns. Desalin Water Treat.

[49] Kuppusamy S (2017). Quercus robur acorn peel as a novel coagulating adsorbent for cationic dye removal from aquatic ecosystems. Ecol Eng.

[50] Öktem YA, Pozan Soylu SG, Aytan N (2012). The adsorption of methylene blue from aqueous solution by using waste potato peels; equilibrium and kinetic studies. J Sci Ind Res (India)..

[51] Guechi E-K, Hamdaoui O (2016). Biosorption of methylene blue from aqueous solution by potato (Solanum tuberosum) peel: equilibrium modelling, kinetic, and thermodynamic studies. Desalin Water Treat.

[52] Hou SX (2013). Adsorption properties of pomelo peels against methylene blue in dye wastewater. Adv Mater Res.

[53] Alrozi, R., Zamanhuri, N. A. & Osman, M. S. Removal of methylene blue from aqueous solution by adsorption onto NaOH-treated rambutan peel Rasyidah. *Bus Eng Ind Appl Colloq* 92–97 (2012).

