# Supplementary material for: Novel approach for effective removal of methylene blue dye from water using fava bean peel waste
Source: Sci Rep. 2020 May 8;10:7824. doi: 10.1038/s41598-020-64727-5 (PMC7210991; doi:10.1038/s41598-020-64727-5)
Supplement: Supplementary file 1 — Supplementary material. [file 41598_2020_64727_MOESM1_ESM.pdf]

Electronic Supplementary Information

Novel approach for effective removal of methylene blue dye from water using fava bean peel waste

Omar S. Bayomie [a,b], Haitham Kandeel [a,c], Tamer Shoeib [a], Hu Yang [d], Noha Youssef [e], Mayyada M.H. El-Sayed [a,*]

[a]Department of Chemistry, American University in Cairo, AUC Avenue, P.O. Box 74, New Cairo 11835, Egypt

[b]Department of Energy and Processes, PSL Research University, Paris, France

[c]Department of Chemistry and Chemical Engineering, École Polytechnique Fédérale de Lausanne, Lausanne, Switzerland

[d]State Key Laboratory of Pollution Control and Resource Reuse, School of the Environment, Nanjing University, Nanjing 210023, P. R. China

[e]Department of Mathematics and Actuarial Science, American University in Cairo, AUC Avenue, P.O. Box 74, New Cairo 11835, Egypt

Email: Mayyada@aucegypt.edu

[*] Mayyada M.H. El-Sayed, email: mayyada@aucegypt.edu

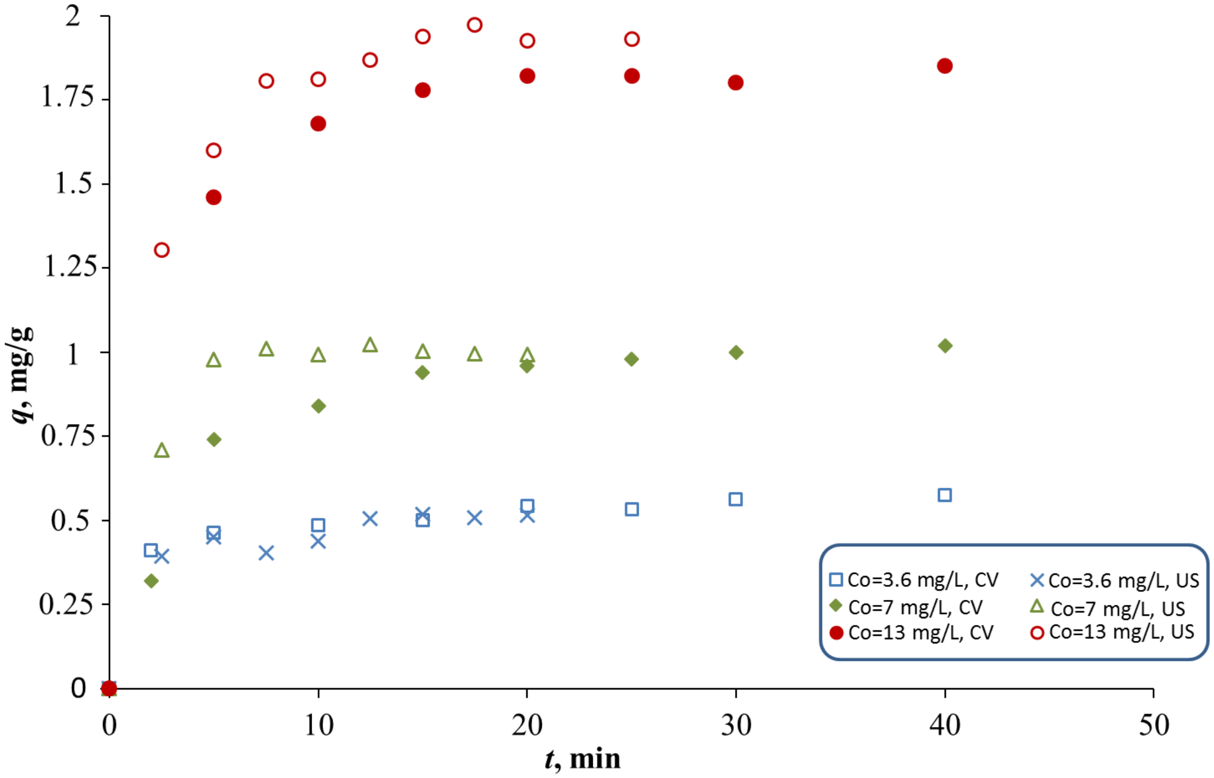

Fig. S1 Uptake profiles for the CV versus the US biosorption of MB onto FBP. Sorption conditions are 5 g/L adsorbent dose and pH 5.8 at low-range of initial concentrations.

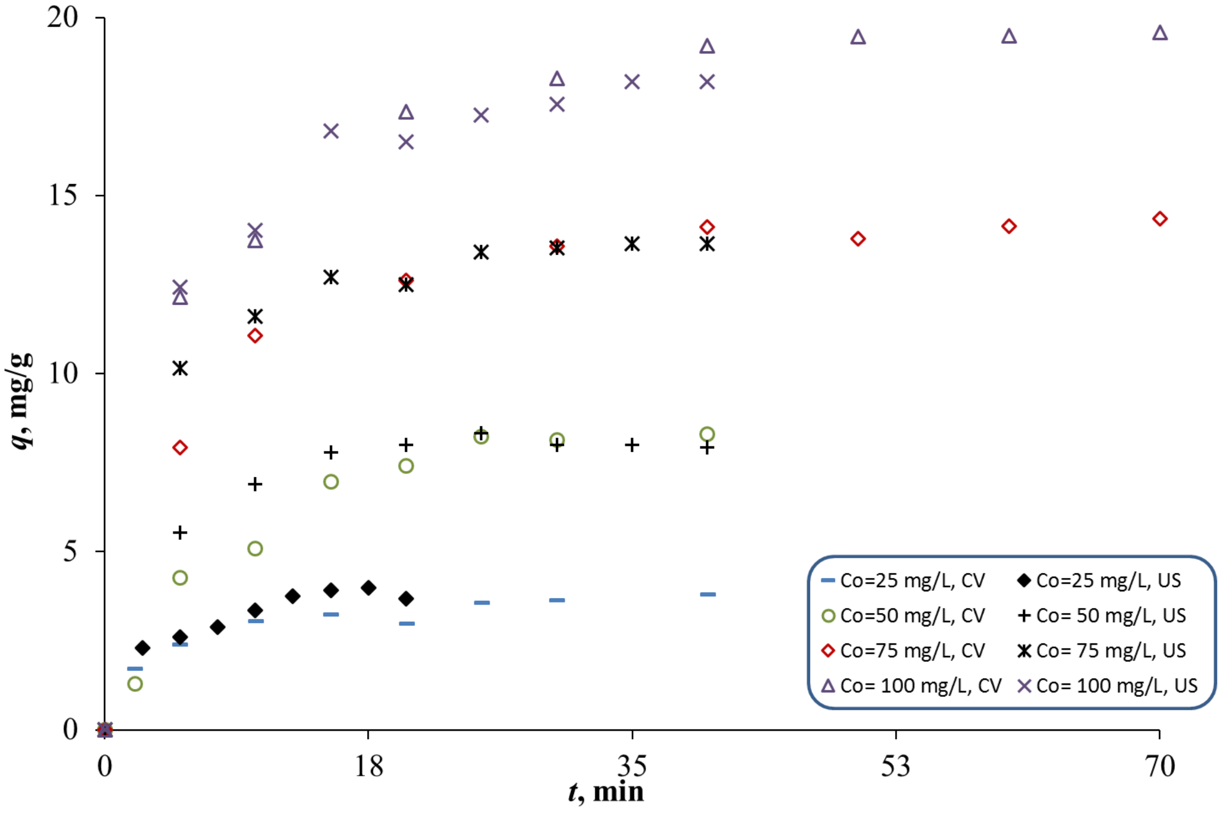

Fig. S2 Uptake profiles for the CV versus the US biosorption of MB onto FBP. Sorption conditions are 5 g/L adsorbent dose and pH 5.8 at high-range of initial concentrations.

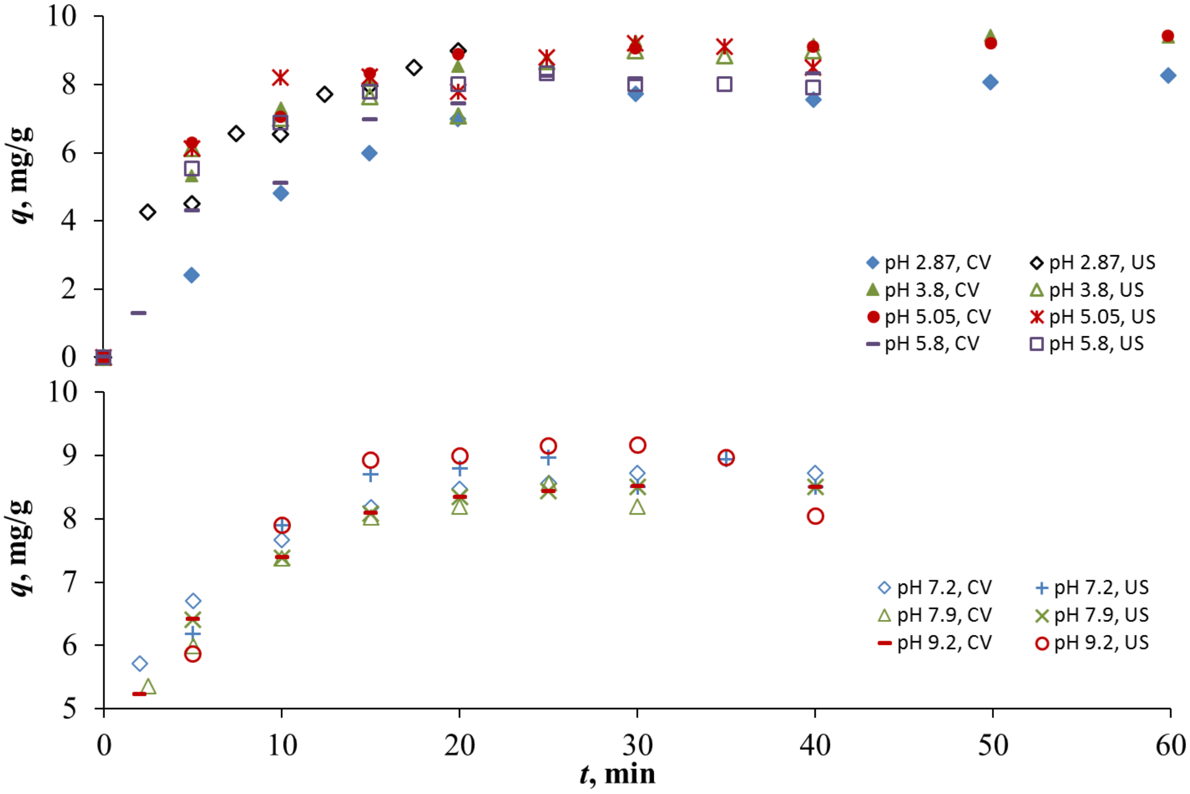

Fig. S3 Uptake profiles for the CV versus the US biosorption of MB onto FBP. Sorption conditions are 5 g/L adsorbent dose and 50 mg/L initial concentration at different acidic (top panel) and basic (bottom panel) pH values.

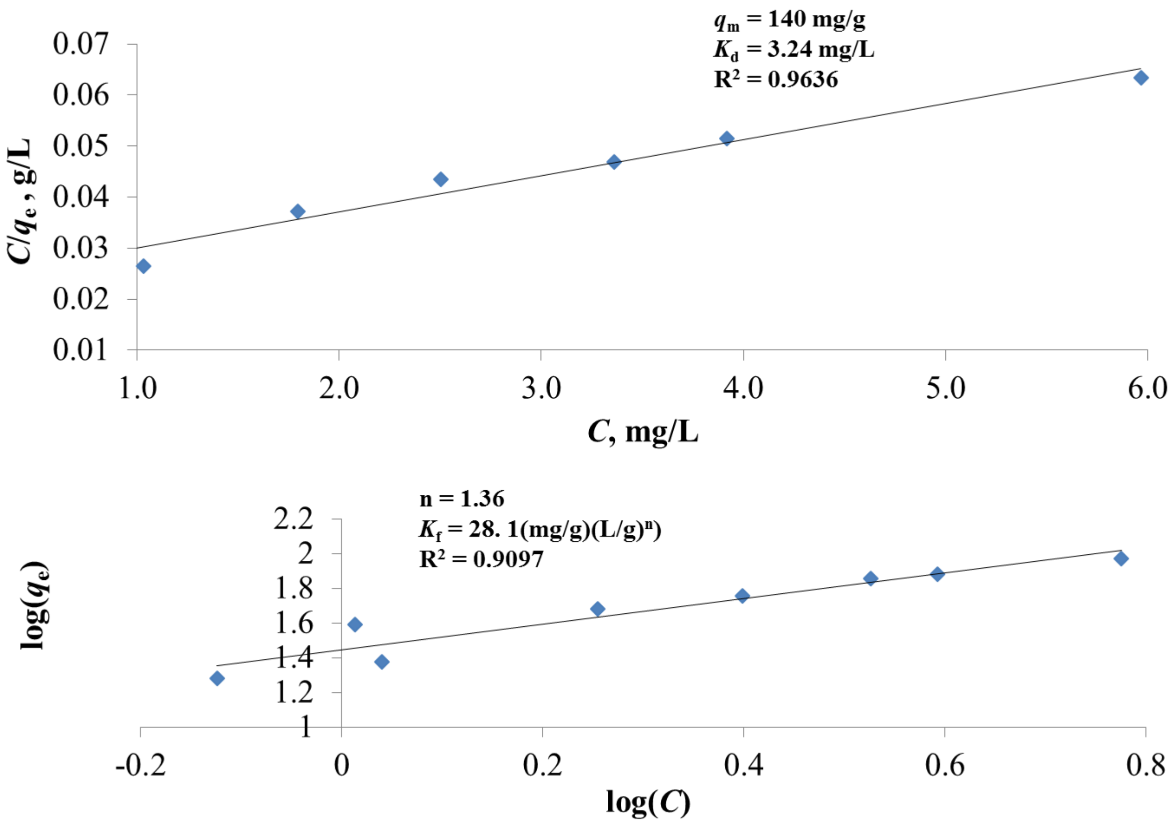

Fig. S4 Linear plots of Langmuir (top panel) and Freundlich (bottom panel) isotherms for the biosorption of MB onto FBP at room temperature (27±2 °C) and pH 5.8.

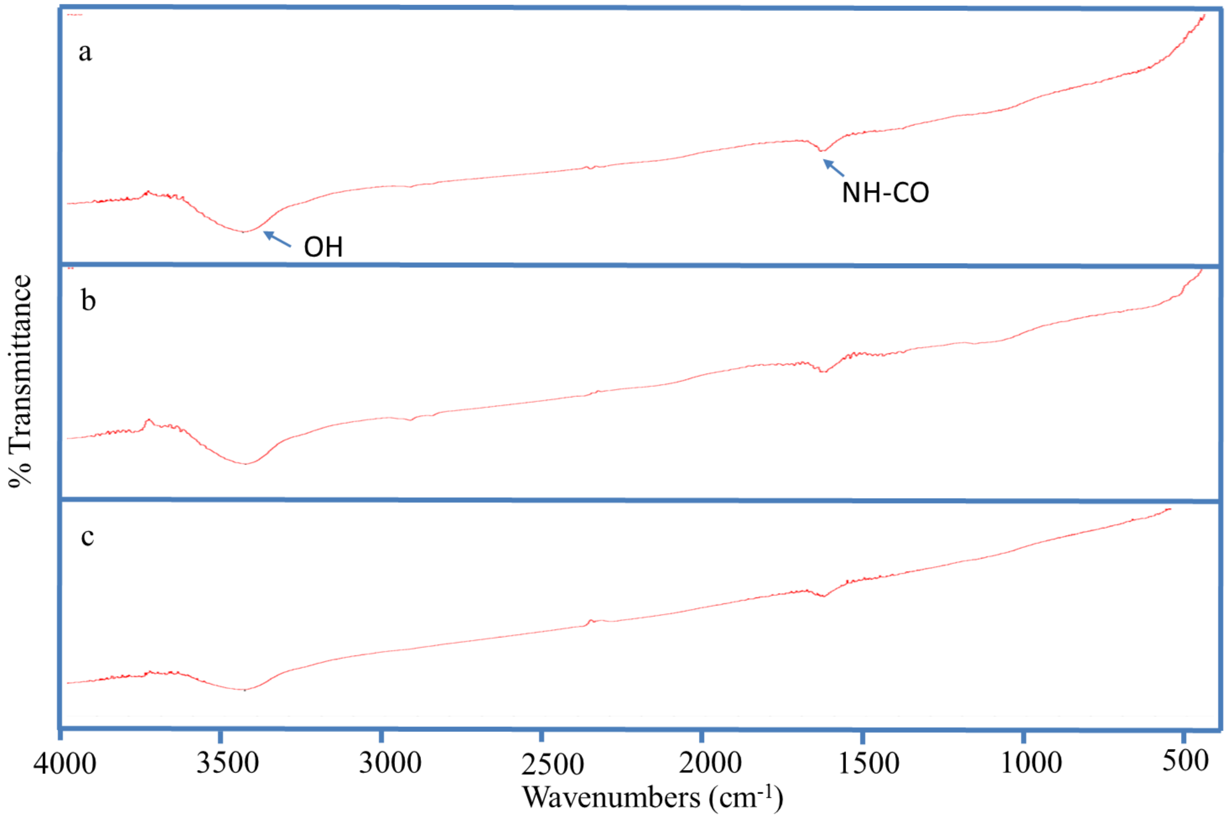

Fig. S5 FTIR spectra for FBP before (panel a) and after CV (panel b) and US (panel c) biosorption.

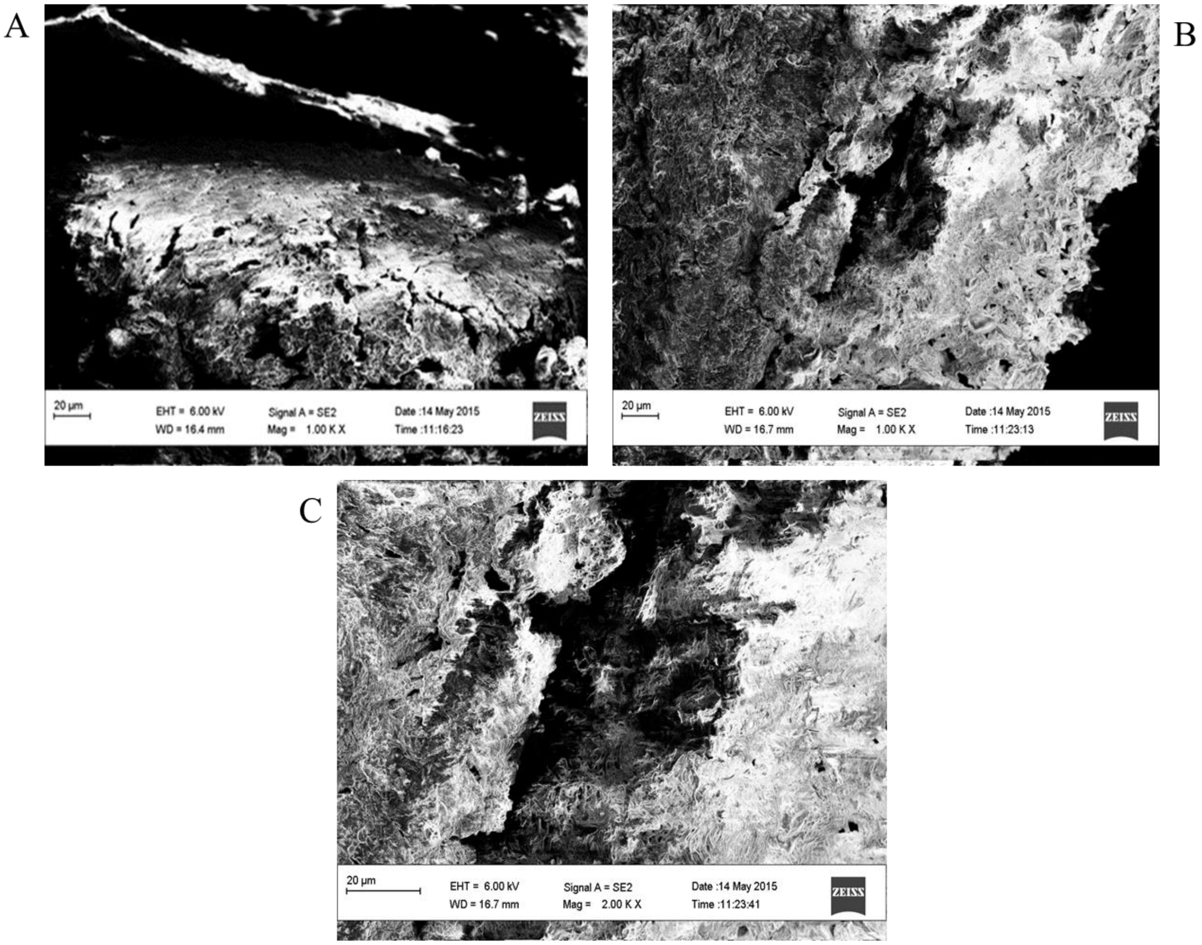

Figure S6 SEM images for FBP before (panel A) and after CV (panel B) and US (panel C) biosorption.

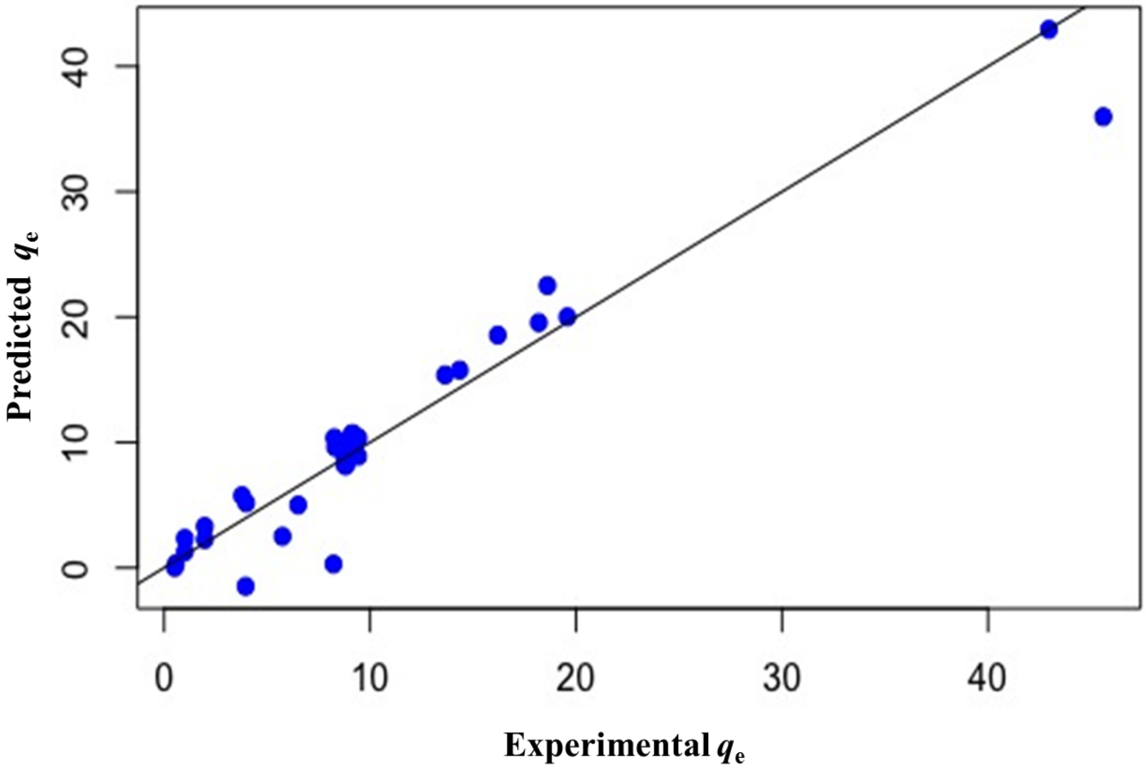

Figure S7 The q_e values as predicted by the linear multiple regression statistical model versus their experimental counterparts.
